# Comparative Assessment of the Remineralization Potential of Five Modern Oral Health Products on Bovine Enamel

**DOI:** 10.3390/jfb17010013

**Published:** 2025-12-25

**Authors:** Aggeliki Lampousi, Dimitrios Dionysopoulos, Razia Z. Adam, Spyros Papageorgiou, Kosmas Tolidis, Robert G. Hill

**Affiliations:** 1 Department of Operative Dentistry, School of Dentistry, Aristotle University of Thessaloniki, 54124 Thessaloniki, Greece; alampousi@dent.auth.gr (A.L.); ktolidis@dent.auth.gr (K.T.); 2 Department of Prosthodontics, Faculty of Dentistry, University of the Western Cape, Parow, Cape Town 7505, South Africa; rzadam@uwc.ac.za; 3 Laboratory of Chemistry-Biochemistry-Cosmetic Science, Department of Biomedical Sciences, University of West Attica, Panepistimioupolis Egaleo Park, 12243 Athens, Greece; spapage@uniwa.gr; 4 Institute of Dentistry, Dental Physical Sciences Unit, Queen Mary University of London, LondonE1 4NS, UK; r.hill@qmul.ac.uk

**Keywords:** bioactive glass, bovineenamel, CPP-ACPF, hydroxyapatite, pH-cycling, stannous fluoride, surface hardness

## Abstract

The purpose of this in vitro study was to evaluate the remineralization effect of five preventive treatments on bovine enamel after artificial caries challenge. Sixty sound bovine incisors were randomly distributed into six experimental groups (*n* = 10). Each group received the following daily preventive treatments for two weeks during pH cycling to assess remineralization efficacy: Group 1—no treatment (control), Group 2—CPP-ACPF treatment, Group 3—fluoride-containing bioactive glass treatment (BioMin™F), Group 4—SnF_2_ treatment, Group 5—toothpaste containing fluoride and hydroxyapatite (HA), and Group 6—toothpaste containing HA. Surface hardness changes were evaluated using a nanoindentation tester. Scanning electron microscopy was used to observe changes in surface morphology, and the mineral composition of enamel in each group was analyzed using energy-dispersive X-ray spectroscopy (EDS). Groups 2, 3 and 6 exhibited the highest surface hardness values after pH cycling, with no statistically significant differences among them (*p* > 0.05), whereas groups 4 and 5 presented significantly lower values (*p* < 0.05). Although all treatments demonstrated significant remineralization potential—resulting in an 18.7–35.2% increase in surface hardness—none of them fully restored the hardness loss caused by demineralization. SEM observations revealed precipitations mainly in groups 2–4 after the treatments. EDS showed a similar elemental composition on enamel across the groups with no differences compared to the control. Additionally, line scans of the Ca and P content from the surface to the deeper layers resulted higher values in the tested groups compared to the control corresponding to the surface hardness values. The tested remineralizing treatments may be useful to limit the demineralizing effect during caries formation.

## 1. Introduction

The balance between tooth demineralization and remineralization is primarily determined by the availability of calcium (Ca^2+^) and phosphate (PO_4_^3−^) ions at the tooth surface [1]. Under normal conditions, the oral fluids surrounding the teeth are saturated or supersaturated with respect to the apatite in enamel and dentin, allowing the tooth tissues to maintain their structural integrity. However, under the influence of various factors that alter the chemical composition of these tissues or the fluids surrounding them, this balance is disrupted. This disruption results in the movement of ions from one phase to another until equilibrium is restored [2]. Among the factors that alter the normal conditions of the oral environment are acids of endogenous origin, which are produced by the microbial plaque during the metabolism of fermentable carbohydrates—mainly derived from food—by the bacteria. Also included are acids of exogenous origin, which come from the consumption of acidic foods and drinks [3]. This loss of hard dental tissues can lead to changes in the physical and mechanical properties of the teeth, alterations in their shape, function, and aesthetics, increased sensitivity, and, in cases of extensive damage, even tooth loss [4].

The saliva mineralization can be accelerated by providing extrinsic sources of Ca^2+^ and PO_4_^3−^, as these ions can facilitate in-depth subsurface remineralization by a higher diffusion gradient [5]. When the pH returns to normal levels, a state of supersaturation is re-established, and the processes are reversed. If this state of supersaturation persists for a period of 3–5 h, it is possible for the complete reuptake of the ions that were lost from the enamel to occur, resulting in the remineralization of the demineralized surface [6].

Nevertheless, the remineralization activity of saliva alone may not be sufficient to restore demineralized lesions, so preventive therapies are recommended to enhance the effectiveness of the remineralization process. Among these therapies, fluoride has traditionally been the primary active ingredient in dental care products designed to prevent the demineralization of tooth tissues [7]. Extensive research supports its effectiveness, particularly through the daily use of fluoridated toothpaste, in significantly reducing demineralization. Although it has been widely recognized that fluoride provides consistent protection against dental decay by enhancing the topical remineralization of early-stage lesions [8,9], the strength of evidence supporting its effectiveness is considered to be of moderate or low certainty [10,11].

Due to this fact, the adoption of new combined strategies to enhance the remineralizing efficacy of fluoride has been recommended [5,12]. Among these, the combination of hydroxyapatite (HA) with fluoride has emerged as a promising alternative. Its superior effectiveness is attributed to the fact that fluoride’s mechanism of action—catalyzing the incorporation of Ca^2+^ and PO_4_^3−^ into the enamel crystal lattice—is typically limited by the availability of these ions in saliva. Consequently, combining fluoride with other calcium- and phosphate-based agents is expected to provide a more effective approach for preventing enamel demineralization and promoting remineralization [13,14]. Nevertheless, when used in a self-administered mode the HA may exhibit the same compliance dependence as products like fluoride [11]. This suggests that fluoride can play a synergistic role in enhancing the performance of other remineralizing treatments [15]. Such treatments may involve the use of other bioactive materials including bioactive glasses [16] and casein phosphopeptide-amorphous calcium phosphate (CPP-ACP) [17], which also have been introduced in various forms for prevention and remineralization of incipient caries or erosive lesions of the teeth. Furthermore, stannous fluoride (SnF_2_) has also been extensively used for caries prevention due to the capability of tin ions to form an acid-resistant protective layer on tooth surface [18].

Therefore, the aim of the present study was to evaluate and compare the effects of five remineralizing treatments on bovine enamel subjected to pH-cycling over a two-week period. The tested treatments involved daily application (for 14 days) of oral health products containing: casein phosphopeptide–amorphous calcium phosphate with sodium fluoride (CPP-ACPF), calcium phosphor-fluoro-silicate glass, stannous fluoride, HA combined with fluoride and HA alone. Remineralization was assessed by measuring changes in surface hardness, while qualitative analysis was performed using scanning electron microscopy (SEM) and energy-dispersive X-ray spectroscopy (EDS). Common approaches for the evaluation of remineralization potential include surface and cross-sectional hardness testing, transverse microradiography, polarized light microscopy, quantitative light-induced fluorescence, and various spectroscopic or diffraction-based techniques. Among these, surface hardness measurements are widely recognized as a reliable and sensitive indirect indicator of mineral gain or loss in enamel. This method offers several advantages for the objectives of our study: it is non-destructive to the subsurface region, provides quantitative data with high reproducibility, and is particularly suitable for evaluating early stages of remineralization where changes primarily occur near the enamel surface.

The first null hypothesis (H_0_1) of the study stated that the tested remineralizing agents would not produce significantly different surface hardness changes compared to the control group, which received no treatment after pH-cycling. The second null hypothesis (H_0_2) was that the tested remineralizing agents would not demonstrate significant differences in their effectiveness at reducing surface hardness loss.

Compared with microbial plaque models, pH-cycling systems provide a more controlled and reproducible method for generating caries-like lesions, as they rely solely on chemically induced demineralization and remineralization rather than bacterial activity. While microbial biofilm models offer greater biological realism by replicating acid production and biofilm behavior, they introduce variability due to differences in bacterial growth and metabolism and require more complex laboratory conditions. In contrast, pH-cycling models allow precise regulation of solution composition, pH, and exposure times, making them well suited for evaluating the mineral effects of preventive agents in a standardized manner, albeit with reduced biological complexity.

## 2. Materials and Methods

### 2.1. Ethical Approval and Randomization Process

This study was approved by the local Ethics and Research Committee (Approval No. 197/14-06-2023) and conducted in accordance with the ethical principles of the 1964 Declaration of Helsinki and its later amendments. Bovine teeth rather than human teeth were used in this study. Whilst bovine teeth have a different prismatic structure from human teeth they generally give more reproducible and consistent results in remineralization studies. Samples were randomly assigned to groups using a computer-generated randomization method (www.random.org website—Randomness and Integrity Services Ltd.; Dublin, Ireland), which minimized selection bias and promoted an even distribution of variables. To prevent subjective bias, evaluators assessing enamel remineralization were blinded to the treatment groups. All samples were subjected to identical handling conditions, including controlled environmental factors and a standardized pH-cycling regimen.

### 2.2. Preparation of the Specimens

Sixty sound bovine incisors were used in the study and were randomly distributed into six experimental groups (*n* = 10). The teeth were free of defects and were stored in a 0.5% chloramine T solution at 6 °C. After the teeth were removed, all residual soft tissue was meticulously cleared away. The external surfaces were then thoroughly cleaned with a slurry composed of pumice and water. Using a water-cooled diamond saw (Isomet, Buehler, Lake Bluff, IL, USA), the crowns were subsequently sectioned from the roots, resulting in specimens approximately 4 mm long, 4 mm wide and 3 mm in height. The 60 tooth crowns were not allowed to be dehydrated and examined by means of optical microscope under 10× magnifications for any surface structural damage or defect. Subsequently, the samples were placed into acrylic resin blocks (NT Newton AYCLIFFE, Antalya, Turkey) with the facial surfaces oriented upward. The exposed enamel was then flattened and polished using a polishing unit (Jean Wirtz TG 250, Düsseldorf, Germany) operating at 200 rpm with continuous water irrigation at 50 mL/min. Polishing was carried out in progressive steps with silicon carbide papers of 600, 800, 1000, and 1200 grit (Apex S system, Buehler, Lake Bluff, IL, USA) for 20 s each to form parallel planar surfaces. After polishing, all specimens were checked for the absence of dentin areas on the polishing surfaces, and immersed in an ultrasonic bath (Euronda Spa, Montecchio Precalcino, Vicenza, Italy) for 5 min to remove any impurities.An adhesive strip (2 mm × 4 mm) was applied to the enamel surface, and the remaining areas of the specimen were coated with an acid-resistant varnish, leaving an exposed treatment area of 8.0 mm^2^ [19].

### 2.3. pH-Cycling Regimen

To induce caries-like lesions on intact bovine enamel blocks of known surface hardness, they were immersed individually in a demineralizing solution (2.2 mM Ca, 2.2 mM P, 50 mM acetate, pH = 4.5 at 37 °C) for 32 h [19], and the surface microhardness was again measured. The 60 specimens were then randomly distributed to six experimental groups (*n* = 10), which were submitted to a pH-cycling model described by previous studies [6,20]. The pH-cycling regimen took 14 days (14 cycles) and included immersion of each specimen individually for 8 h into 20 mL of the demineralization solution described above at 37 °C, and for 16 h into 20 mL of a remineralization solution (1.5 mM Ca, 0.9 mM P, 130 mM KCl, 20 mM buffer TCP, pH = 7.0 at 37 °C). Two times a day (9:00 and 21:00 h), the blocks were rinsed with deionized water for 5 s and smeared for 3 min with one of the tested slurries under agitation. After the treatments, the blocks were rinsed again for 5 s and immersed inthe remineralizing solution. Every three days, the de- and remineralizing solutions were replaced by fresh ones. After 14 cycles, the enamel remineralization capacity of each treatment was evaluated by measuring the surface hardness.During pH-cycling procedureastandardized water-rinsing protocol was used, so that no additional cleaning steps were applied, and any remaining trace amounts of slurry or paste were considered negligible and uniformly distributed among groups.

### 2.4. Experimental Groups of the Study

Each experimental group of the study (*n* = 10) received one of the following treatments during pH-cycling regimen that was described above:

Group 1 (control group): no treatment was received.

Group 2: the specimens were smeared twice daily for two weeks with GC MI Paste Plus (GC Corp., Tokyo, Japan) slurry, which contained casein phosphopeptide—amorphous calcium phosphate and 900 ppmF^−^(CPP-ACPF).

Group 3: the specimens were smeared twice daily for two weeks with BioMin^TM^F Tooth Mousse Plus (Cera Dynamics Ltd., Fenton, UK) slurry, which contains a fluoride-containing BAG (calcium phospho-fluoro-silicate).

Group 4: the specimens were smeared twice daily for two weeks with Emofluor^®^ (Dr. Wild & Co., AG, Muttenz, Switzerland) slurry, which contained 0.4% stannous fluoride (SnF_2_).

Group 5: the specimens were smeared twice daily for two weeks with SensiTeeth Epismalto (Frezyderm S.A., Athens, Greece) slurry, which contains 8% hydroxyapatite and fluoride (1450 ppmF^−^).

Group 6: the specimens were smeared twice daily for two weeks with Bite & Brush slurry (Kindbrush, Cape Town, South Africa), which contains 10% hydroxyapatite.

The slurries of the products were made by diluting 1 part of the tested product in 3 parts of deionized water, and applied directly on the surface of enamel with a plastic syringe [21]. The compositions of the tested commercial products are presented in Table 1.

### 2.5. Evaluation of Surface Hardness

Surface hardness of enamel was assessed at three different points intime: T_1_—initially (intact enamel), T_2_—after caries-like lesions formation, and T_3_—following pH-cycling regimen, using a nanoindentation tester (Hit 300, Anton Paar TriTec SA, Corcelles, Switzerland). Poisson’s ratio of the enamel was assumed to be 0.25 in this study [22]. A Berkovich diamond indenter was used, applying a maximum load of 40 mN with a loading time of 30 s, an approach speed of 4000 nm/min, and an acquisition rate of 10 Hz under a quadratic loading profile. Ten indentations were made on the surface of each specimen, spaced 100 μm apart. This design ensured that indentations remained within an area of comparable surface condition while avoiding interaction effects between adjacent plastic zones. The mean indentation hardness values, expressed in GPa, were calculated using Indentation Software Version 10.2.4 (Anton Par TriTec SA, Corcelles, Switzerland).

### 2.6. Assessment of Remineralization Using SEM-EDS Analysis

Scanning electron microscopy (JEOL Ltd., JSM-840, Tokyo, Japan) was used to evaluate the effects of the tested remineralization treatments on demineralized enamel. Following the final surface hardness measurements at the end of the pH-cycling procedure, three representative specimens from each experimental group were randomly selected for SEM analysis. In particular, the tooth crown specimens were sectioned perpendicularly through the center of the enamel surface. One half of each cut specimen was then mounted on aluminum stubs with the labial surface facing upward, while the other half was mounted with the sectioned surface facing upward. The specimens were sputter-coated with carbon to a thickness of approximately 200 Å in a vacuum evaporator under low vacuum and examined at an accelerating voltage of 20 kV. For the first half-specimen, four secondary electron imaging (SEI) micrographs were captured at ×1000 magnification, one from each quadrant of the labial surface of enamel to evaluate morphological changes after treatment. For the second half-specimen, three backscattered electron (BSE) micrographs were obtained at ×50 magnificationfrom different areas of the sectioned surface: one in the center and two at least 500 μm apart. Elemental qualitative analysis was performed using EDS (JEOL Ltd., Tokyo, Japan), and line-scans were carried out to monitor fluctuations in Ca and P composition, providing an estimate of demineralization depth. All images were independently evaluated by two blinded examiners.

### 2.7. Statistical Analysis

Statistical analyses were performed using SPSS Statistics 25.0 software (IBM Corp., Chicago, IL, USA). Sample size was determined through a power analysis conducted with G*Power 3.1.9.4, assuming 80% power and a significance level of 0.05. Data normality and homogeneity of variances were evaluated using the Shapiro–Wilk and Levene tests, respectively. A two-way repeated-measures ANOVA (factors: treatment group × time of measurement) was applied to assess the effects of different remineralizing treatments and demineralization conditions on enamel surface hardness. Post hoc pairwise comparisons were conducted with Bonferroni correction. In all the analyses, the level of significance was set at a = 0.05.

## 3. Results

**Figure 1:**
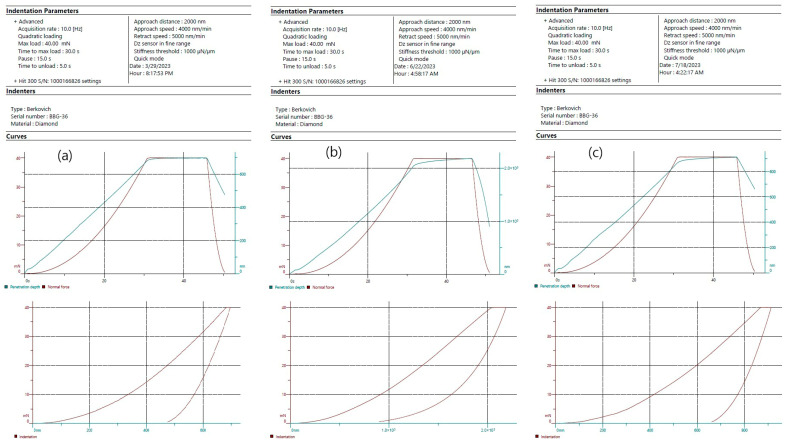
Representative nanoindentation load–displacement curves of a specimen treated with CPP-ACPF, illustrating the typical elastic–plastic response recorded for enamel under the Berkovich indenter, for each time point of measurements. Also, the indentation depth is shown for each curve, which was appropriate for enamel to minimize substrate effects and ensure that the deformation zone remained within the treated enamel layer. (**a**): Baseline measurement; (**b**): Measurement after demineralization; (**c**): Measurement after CPP-ACPF treatment.

Means and standard deviations of the enamel surface hardness of the experimental groups of the study in GPa are presented in Table 2. Also, means and standard deviations of the elastic modulus of the enamel of each experimental group in GPa are presented in Table 3. In Figure 1, representative nanoindentation load–displacement curves of a specimen treated with CPP-ACPF are illustrated for each time point of measurements.

Following immersion of the specimens in the demineralizing solution for inducing caries-like lesions, enamel surface hardness was decreased to less than 20% of the initial values. All tested remineralizing treatments showed beneficial effects on enamel surface hardness compared to the control group (*p* < 0.05). Specifically, groups 2, 3 and 6 exhibited the highest surface hardness after the pH-cycling with no statistically significant differences among them (*p* > 0.05), whereas groups 4 and 5 presented significant lower surface hardness values (*p* < 0.05). The percentage of the final surface hardness relative to the initial values of intact enamel ranged from 38.4% to 54.9%, compared with only 19.7% for the untreated group. This indicates that the remineralizing treatments improved surface hardness by 18.7–35.2%. However, none of the treatments fully restored the hardness loss caused by the demineralization.

**Figure 2:**
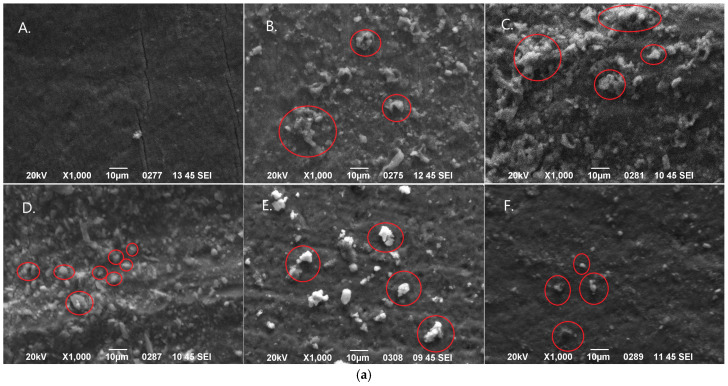

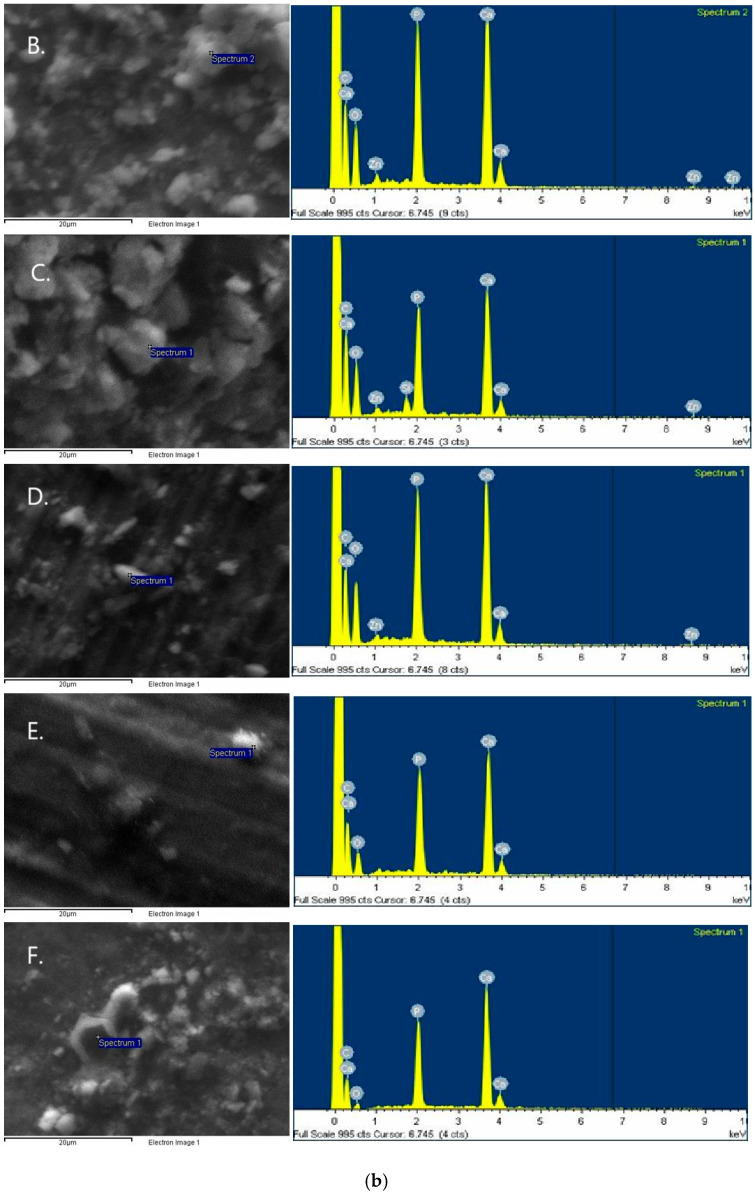
(**a**). Representative SEΙ micrographs showing the labial enamel surface after treatments in each experimental group at ×1000 magnification. Red circles indicate precipitates deposited during the treatments. (**b**). Representative BSE images showing the formed precipitations after the treatments in each tested group at ×3000 magnification. EDS spectra beside the images indicate the elemental composition of the examined precipitations. (**A**). control group; (**B**). CPP-ACPF treatment; (**C**). calcium fluoro-phospho-silicate glass treatment; (**D**). stannous fluoride treatment; (**E**). treatment with hydroxyapatite + fluoride; and (**F**). treatment with hydroxyapatite.

**Figure 3:**
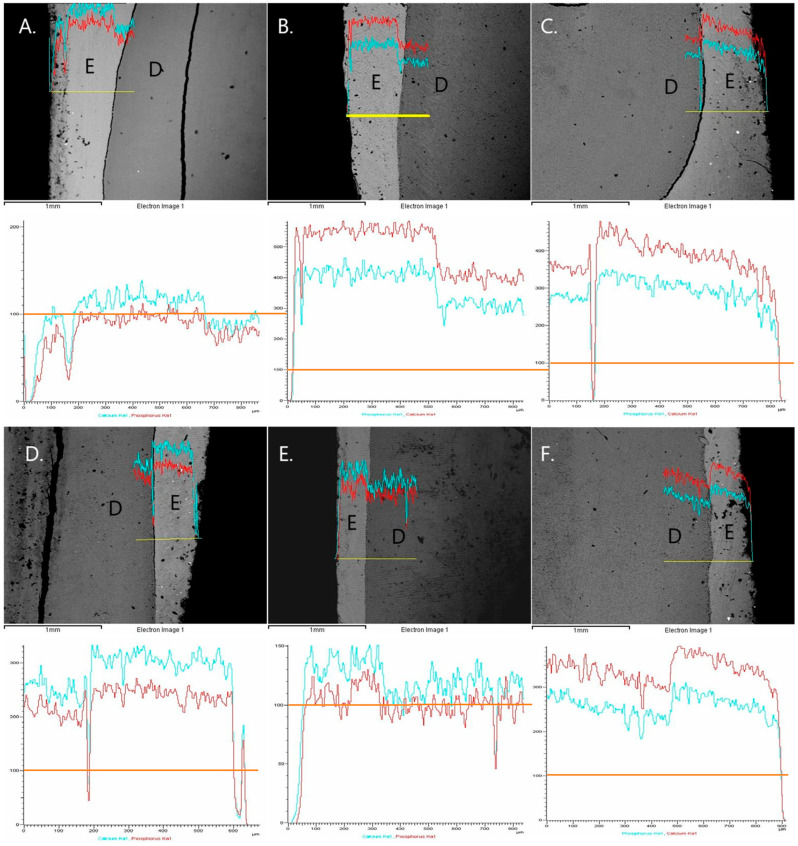
Representative BSE micrographs of the sectional surface after treatments in each experimental group (×50 magnification). Red lines indicate fluctuations in Ca content at different enamel depths, while blue lines represent P content. E: enamel, D: dentin. The orange line indicates the value 100, which is near the control group values. (**A**). control group; (**B**). CPP-ACPF treatment; (**C**). calcium phosphor-fluoro-silicate glass treatment; (**D**). stannous fluoride treatment; (**E**). treatment with hydroxyapatite + fluoride; and (**F**). treatment with hydroxyapatite.

Representative SEM images (×1000 magnification) of the labial enamel surface following the treatments from each experimental group are presented in Figure 2a. Moreover, representative SEM images with higher magnification (×3000) showing the microstructure of the precipitations that were formed after the treatments are presented in Figure 2b. Precipitations were observed in groups 2–4 after the treatments. The EDS spectra of the elemental composition of the precipitations that were detected in each image are also shown beside the images in Figure 2b. Additionally, representative BSE micrographs of the sectional surfaces of the specimens after treatment in each experimental group, at ×50 magnification, are shown in Figure 3. Line scans of the Ca and P content from the surface to the deeper layers correlated with surface hardness results: the higher the Ca and P content, the greater the surface hardness. As it was previously mentioned, all specimens were sputter-coated with a thin carbon layer prior to SEM/EDS analysis to ensure adequate surface conductivity and imaging quality. Because this carbon coating introduces an exogenous and uniform carbon signal over the entire specimen surface, the carbon peak detected by EDS does not originate from the enamel or from the applied treatments. For this reason, carbon was intentionally excluded from the elemental analysis.

## 4. Discussion

Based on the results of the present study H_0_1 stated that the tested remineralizing agents would not produce significantly different surface hardness changes compared to the control group, which did not receive any treatment following pH-cycling, was rejected. Likewise, H_0_2, stated that the tested remineralizing agents would not demonstrate significant differences in their effectiveness at reducing surface hardness loss, was also rejected. This is consistent with previous studies, where there was evidence for the effectiveness of the tested remineralizing agents. More specifically, for treatments with CPP-ACPF [13,23], bioactive glass [24,25], SnF_2_ [14,26], and HA alone [9,27] or combined with fluoride [7,28], an increase in enamel surface hardness has been reported after demineralization challenges. The discrepancies among the experimental groups in the remineralizing potential may be attributed to differences in the mechanism of action, concentration, product form, amount of applied product or stability of the remineralization agents on enamel surface.

In the current study, GC MI Paste Plus, which contains CPP-ACPF, increased enamel surface hardness from 0.52 to 2.35 GPa (more than a threefold increase) after a two-week treatment. ACPF is formed as a result of ACP taking up fluoride. This reduces the F ion concentration and stabilises the ACP. The ACPF has a 19F signal close to that of fluorapatite [29]. Casein phosphopeptides (CPPs) have the remarkable ability to interact with both positively and negatively charged ions, such as Ca^2+^, PO_4_^3−^, and F ions, leading to the formation of complexes like CPP-ACPF [24]. The Recaldent™ technology, which utilizes CPP-ACP, offers the most robust clinical evidence supporting its efficacy in tooth remineralization [23]. Derived from milk proteins, CPPs form nanocomplexes that stabilize amorphous calcium phosphate (ACP) through multiphosphorylated peptide structures. This mechanism maintains a steady reservoir of bioavailable Ca^2+^ and PO_4_^3−^ ions in solution, enabling their diffusion into enamel and promoting the remineralization of hypomineralized regions [30]. Furthermore, CPP-ACP enhances the ionic concentration in saliva, delaying premature precipitation and enabling deeper ion diffusion into subsurface enamel lesions [23].Moreover, CPPs have been shown to significantly inhibit the adhesion of Streptococcus mutans to the salivary pellicle, which may decrease the cariogenic potential of microbial plaque [31].

The mechanism underlying the action of CPP-ACPF involves multiple interrelated processes that collectively enhance its protective and restorative effects on tooth surfaces. Initially, CPP-ACPF increases the density of Ca-binding sites on enamel, thereby reducing the continuous loss of calcium through diffusion. The incorporated ACP functions as a dynamic reservoir, buffering free Ca^2+^ and PO_4_^3−^ and maintaining a state of supersaturation at the enamel interface. This supersaturated environment is critical for minimizing demineralization while promoting remineralization of tooth structure [32]. Additionally, the application of CPP-ACP facilitates the deposition of a mineralized layer over the enamel surface [33]. Although this layer is not completely uniform, it exhibits globular formations that adhere to and partially occlude interprismatic spaces, as observed under SEM in the present study (Figure 1) and aligns with the observations of other similar studies [13,34]. Robust evidence in the literature indicates that CPP-ACP exhibitsa significant remineralizing effect in both in vitro and in vivo studies, suggesting it may be a more promising agent than fluoride-based products for the clinical management of early enamel carious lesions [35]. Barrera-Ortega et al. [13] reported a large improvement in surface hardness (from 281.0 ± 04.1 to 515.2 ± 10.7 VHN) of a CPP-ACPF treatment after pH-cycling regimen similar to the current study. Also, Kaur et al. [23] found a 35.7% decrease in enamel surface hardness compared to the intact enamel, while in the present study this decrease was up to 46%.

BioMin^TM^F Tooth Mousse Plus, which contains fluoro-calcium-phospho-silicate glass particles, also significantly increased enamel surface hardness (from 0.62 to 2.25 GPa) after two weeks of treatment to a level similar to that achieved with GC MI Paste Plus. BioMin™F is a specially engineered calcium phospho-fluoro-silicate designed as a functional additive for toothpaste formulations. Compared with other bioactive glasses, it contains a higher concentration of phosphate ions, which accelerates the nucleation and growth of apatite and increases the overall amount formed [36]. The elevated phosphate level however reduces the pH increase during the glass’s dissolution process [37]. This pH increase is an important and unique feature of bioactive glass toothpastes. The incorporation of small quantities of fluoride further enhances the speed of apatite formation, promoting the development of fluorapatite rather than hydroxycarbonated apatite formed with conventional bioactive glasses. Nevertheless, as the fluoride concentration within the glass increases, the rise in pH becomes progressively smaller, which can favor the precipitation of CaF_2_ instead of fluoroapatite [38] (a phase known for its superior resistance to erosive challenges).

Earlier research has shown that bioactive glasses (BAGs) improve surface hardness and encourage the development of a more uniform and durable mineralized layer than fluoride treatments alone. Thoutam et al. [25] demonstrated that a treatment with a fluoride-containing BAG increased surface hardness from 59.7 ± 3.03 to 358.9 ± 37.6 VHN. Findings from both qualitative observations and quantitative measurements consistently demonstrate that BAGs provide more effective remineralization than fluoride as well as other commonly used agents, including CPP-ACP and HA [39]. In the present study, using BioMin™F, a protective layer of apatite precipitations was observed through SEM-EDS analysis (Figure 1). This is consistent with earlier studies that reported similar SEM observations [25,36]. Particles abundant in Ca and P covered nearly the entire enamel surface. Additionally, an extra peak of silicon (Si) was detected, identifying the presence of bioactive glass. Bioactive glasses undergo dissolution upon contact with tooth surfaces and saliva;this is accelerated in acidic conditions, similar to thoseinduced during caries formation, where this process is more rapid. As they break down, they release Ca^2+^ and PO_4_^3−^ ions, which helpelevate the local pH and supportthe repair of subsurface enamel that has been demineralized by acidic by-products of oral bacteria [40]. Through a sequence of chemical reactions, a hydroxycarbonate apatite layer gradually forms and chemically integrates with the enamel surface [41]. This newly formed layer typically requires several hours to develop fully and acts as a protective shield against erosive challenges, thereby improving the tooth’s resistance to further mineral loss [40]. In the case of BioMin™F the apatite formed is a fluorapatite, which is much more acid-resistant. This mechanism may also have contributed to the favorable results that were obtained in the current investigation following BAG treatment.

Stannous fluoride, which is included in Emofluor^®^, also induced remarkable remineralization effect on enamel leading to increase in mean surface hardness from 0.68 to 1.76 GPa (48.7% recovery) after a two-week treatment. However, the findings of the present study indicated that the remineralization effect of the SnF_2_ treatment was lower than that observed with the CPP-ACPF treatment. This observation is in agreement with previous research reporting significantly greater remineralization and surface microhardness recovery with a CPP-ACP-containing toothpaste compared to two SnF_2_-based dentifrices [14]. Conversely, an earlier study demonstrated that the combination of SnF_2_ and CPP-ACP produced a more pronounced suppression of acidogenic and aciduric bacteria, along with a significant 72% reduction in enamel demineralization [42].

The long-term stability, bioavailability and anticariogenic activity of SnF_2_ in dentifrices have been well documented in previous studies and are associated with the prevention of tooth caries [43,44,45]. The proposed mechanism of action involves the high affinity of tin ions (Sn^2+^) for enamel mineral components, resulting in the formation of a protective surface layer that can extend to a depth of approximately 20 μm [46]. This layer is believed to confer prolonged protection against cariogenic challenges. Although SnF_2_ is effective, it does have certain clinical drawbacks, such as a metallic taste, the possibility of tooth staining, and a potential reduction in taste sensitivity with long-term use [47]. Similar SEM observations after SnF_2_ treatment have been demonstrated in previous investigations [36].

In the current study, two remineralizing treatments containing HA, one combined with fluoride and the other containing HA alone, were evaluated. Both treatments effectively remineralized the demineralized enamel surfaces resulting in surface hardness recovery of 38.4% and 54.9%, respectively. This discrepancy between the two treatments was unexpected, as the combination of HA with F was intended to enhance the remineralizing potential of this toothpaste. In fact, the interaction between HA and F can lead to the formation of fluorapatite (FA), which is considerably more stable than HA. However, FA can be formed only if there are free fluoride ions but not if the fluorine is present as sodium monofluorophosphste (MFP), in which the F is covalently bonded to P, as in the case possibly of SensiTeeth. But F cannot be released from MFP without the action of alkaline phosphatase (ALP) enzyme, which hydrolyzes the P–F bond [48,49]. Nevertheless, in this in vitro study ALP was absent, as there would be in saliva, so little or no fluoride was available from this product. Consequently, the current experimental protocol may underestimate the remineralization potential of the SensiTeeth toothpaste, which could explain the lower values observed compared with the HA-based product (Bite & Brush). ALP was not used in the study since the manufacturer did not disclose the presence of MFP in the original product description. Furthermore, an interesting result was that the HA toothpaste containing fluoride was less effective and not equally effective than the HA-only toothpaste in terms of SH recovery. This may be attributed to differences in the form or amount of HA present (8% in the SensiTeeth compared to 10% in the Bite & Brush). Amaechi et al. [27] found differences in the ability of HA toothpastes to enhance remineralization of early enamel caries lesions ranged from 19.50 to 39.81%, which is similar to those reported in the current research for SensiTeeth (38.4%).

Hydroxyapatite has the chemical formula Ca_10_(PO_4_)_6_(OH)_2_. It represents the principal calcium phosphate mineral and the main inorganic constituent of all human hard tissues, including teeth. In addition, owing to its bioactive and biocompatible nature, it exhibits significant remineralizing and preventive potential, attributed to its capacity to adsorb effectively onto tooth tissues, microbial plaque and bacterial cells, thereby filling enamel interprismatic spaces [50]. A key advantage of HA is its non-toxic nature, rendering it exceptionally safe for daily use [51]. In contrast, excessive and chronic fluoride intake has been associated with increasedproduction of reactive oxygen species, potentially leading to organ toxicity, impaired immune function and, in children, fluorosis, neurodevelopmental effects and cognitive impairment [52].

With respect to the mechanism of action, HA particles adhere to the enamel surface, via carboxylated cellulose or another carboxylic acid-containing polymer, where they serve as a reservoir for Ca^2+^ and PO_4_^3−^. The carboxylate groups serve to stick the HA particles to the tooth surface via Ca ion chelation much like a GIC. During acidic episodes, these ions are liberated, helping to keep the oral environment supersaturated with enamel-building minerals. This ionic release also contributes to buffering the pH, thereby creating favorable conditions for remineralization [53]. It is also claimed that HA forms a protective layer on the tooth surface that inhibits the adhesion and proliferation of cariogenic bacteria, reduces biofilm formation, and protects against further demineralization [5]. Furthermore, HA contributes to neutralizing acidity within the oral cavity, creating a more alkaline environment favorable to remineralization [9].

The literature indicates a non-significant trend favoring HA toothpastes over fluoride-based ones. This suggests that HA products may serve as a viable option for slowing caries development and enhancing the remineralization of early enamel lesions, especially in young individuals with elevated caries risk [9]. These observations are consistent with the results of the current study as well as prior investigations [5,54], which have reported a greater remineralizing effect of HA-containing toothpastes compared to those with fluoride. However, clinical evidence supporting HA monotherapy remains insufficient [51]. Moreover, while some studies have shown that combining HA with fluoride yields a positive effect on the remineralization of early carious lesions, this combination has not consistently demonstrated superiority over other therapies, such as CPP-ACPF or even fluoride alone, in certain cases [7]. These findings are in agreement with the results of the present investigation. It has been proposed that, similar to fluoride, HA may have limited ability to diffuse into the deeper regions of demineralized enamel due to the presence of a highly mineralized surface layer [53]. Although interactions between HA and fluoride compounds have been observed, this interaction does not appear to enhance their surface attachment compared to the application of fluoride alone [55].

The study was conducted under controlled laboratory conditions, which do not fully replicate the complex biological environment of the oral cavity. The use of bovine enamel is another limitation of this study. Although bovine enamel is commonly used as a substitute for human enamel in laboratory studies due to its availability and comparable structure, it differs in fluorine content in the enamel surface, in prism structure, porosity, and permeability. These differences may influence the remineralization response. Additionally, the two-week treatment and pH cycling period may not adequately represent long-term clinical conditions. The durability and stability of the remineralized layer over extended periods remain uncertain. Also, including a sound (non-demineralized) enamel control could offer additional contextual insight into the magnitude of mineral recovery. Finally, the lack of biological factors such as salivary proteins, like alkaline phosphatase, pellicle formation, and bacterial biofilm activity, may have affected the extent and pattern of mineral deposition.

## 5. Conclusions

Within the limitations of this in vitro study, all tested preventive treatments demonstrated the ability to promote enamel remineralization after an artificial caries challenge. Treatments containing CPP-ACPF, fluoro-calcium phospho-silicate, and HA alone were the most effective in increasing surface hardness, showing comparable remineralization efficacy, while SnF_2_ and HA combined with F presented lower surface hardness recovery. However, none of the treatments completely restored enamel hardness to its original state. These findings suggest that the evaluated remineralizing agents may help reduce enamel demineralization and could serve as useful adjuncts in caries prevention strategies. Further in vivo studies are recommended to confirm their clinical effectiveness.

## Figures and Tables

**Table 1:** The composition of the tested commercial products of the study and their active agents according to manufacturers.

Product	Form	Composition	Active Agents	Manufacturer
GC MI Paste Plus	paste	Pure water, glycerol, Recaldent (CPP-ACP), D-sorbitol, CMC-Na, propylene glycol, silicon and titanium dioxide, xylitol, phosphoric acid, flavour, NaF (900 ppmF^−^), sodium saccharin, ethyl-, propyl-, butyl-, p-hydroxybenzoate	Recaldent (CPP-ACP) + NaF (900 ppmF^−^)	GC Corp., Tokyo, Japan
BioMin^TM^F Tooth Mousse Plus	paste	Glycerin, silica, PEG 400, sodium lauryl sulphate, titanium dioxide, aroma, carbomer, potassium acesulfame, fluor- calcium-phospho-silicate: 36–40 mol% SiO_2_, 22–24 mol% Na_2_O, 28–30 mol% CaO, 4–6 mol% P_2_O_5_, 1.5–3.0 mol% CaF_2_	Bioactive glass (fluor-calcium phosphor-silicate) + CaF_2_	Biomin Technologies Ltd., London, UK
Emofluor^®^	gel	Aqua, glycerin, propylene glycol, PEG-40-hydrogenated castor oil, cellulose gum, PEG-8, phosphocolamine, aroma, 0.4% SnF_2_ (1000 ppmF^−^), sodium saccharin	SnF_2_ (1000 ppmF^−^)	Dr. Wild & Co. AG, Muttenz, Switzerland
SensiTeeth Epismalto	paste	8% hydroxyapatite, 1450 ppmF^−^, myrrh, sage, xylitol, bisabolol, mild surfactants	8% hydroxyapatite+ 1450 ppmF^−^	Frezyderm S.A., Athens, Greece
Bite & Brush	tablets	10% hydroxyapatite, xylitol, zinc citrate, calcium carbonate, sodium cococyl glutamate, peppermint, spearmint oil	10% hydroxyapatite	Kindbrush, Cape Town, South Africa

CaF_2_: calcium fluoride; CaO: calcium oxide; CMC-Na: sodium carboxymethyl cellulose; CPP-ACP: casein phosphopeptide-amorphous calcium phosphate; Na_2_O: sodium oxide; PEG: polyethylene glycol; P_2_O_5_: phosphorus pentoxide; SiO_2_: silicon dioxide; SnF_2_: stannous fluoride.

**Table 2:** Means and standard deviations of surface hardness (GPa), at three different points of time: initially (intact enamel), after caries-like lesions formation, and following the pH-cycling regimen. The percentage (%) of the final surface hardness after the treatments and pH-cycling in comparison with the initial surface hardness of the enamel (intact) of each experimental group is also presented for each experimental group.

Group	Treatment	Active Agent	Initial SH	SH After 32 h Demineralization	SH After pH-Cycling and Treatments	% Recovery of the Initial SH
1	Control	-	4.11 (0.29) ^Aa^	0.71 (0.25) ^Ab^	0.81 (0.23) ^Ab^	19.7%
2	GC MI Paste Plus	CPP-ACP + F	4.35 (0.34) ^Aa^	0.52 (0.24) ^Ab^	2.35 (0.54) ^Bc^	54.0%
3	BioMin^TM^F Tooth Mousse Plus	Calcium fluoro- phospho-silicate glass	4.17 (0.85) ^Aa^	0.62 (0.23) ^Ab^	2.25 (0.44) ^Bc^	53.9%
4	Emofluor^®^	SnF_2_	3.61 (0.44) ^Aa^	0.68 (0.26) ^Ab^	1.76 (0.34) ^Cc^	48.7%
5	SensiTeeth Epismalto	HA + F	4.30 (0.20) ^Aa^	0.62 (0.27) ^Ab^	1.65 (0.12) ^Cc^	38.4%
6	Bite & Brush	HA	4.08 (0.94) ^Aa^	0.63 (0.18) ^Ab^	2.24 (0.15) ^Bc^	54.9%

SH: surface hardness. Same uppercase superscripts in columns indicate no significant differences among the treatments (*p* >0.05). Same lowercase superscripts in rows indicate no significant differences among the times of measurements (*p* > 0.05).

**Table 3:** Means and standard deviations of elastic modulus (GPa), at three different points of time: initially (intact enamel), after caries-like lesions formation, and following the pH-cycling regimen.

Treatment	Initial Elastic Modulus	Elastic Modulus After 32 h Demineralization	Elastic Modulus After pH-Cycling and Treatments
Control	82.45 (5.26) ^Aa^	27.89 (7.11) ^Ab^	31.93 (8.24) ^Ab^
GC MI Paste Plus	92.47 (3.13) ^Aa^	10.78 (3.81) ^Bb^	55.86 (4.95) ^Bc^
BioMin™F Tooth Mousse Plus	86.72 (7.49) ^Aa^	24.92 (12.00) ^Ab^	51.61 (7.33) ^Bc^
Emofluor^®^	79.20 (7.26) ^Aa^	27.56 (10.43) ^Ab^	42.63 (15.18) ^Cc^
SensiTeeth Epismalto	86.89 (1.64) ^Aa^	15.32 (8.10) ^Bb^	42.90 (10.50) ^Cc^
Bite & Brush	86.15 (8.11) ^Aa^	23.83 (8.62) ^Ab^	52.69 (22.59) ^Bc^

Same uppercase superscripts in columns indicate no significant differences among the treatments (*p* > 0.05). Same lowercase superscripts in rows indicate no significant differences among the times of measurements (*p* > 0.05).

## Data Availability

The original contributions presented in the study are included in the article, further inquiries can be directed to the corresponding author.
